# Comparison of volatile anesthetic-induced preconditioning in cardiac and cerebral system: molecular mechanisms and clinical aspects

**DOI:** 10.1186/s40001-018-0308-y

**Published:** 2018-02-20

**Authors:** Shasha Chen, Christopher Lotz, Norbert Roewer, Jens-Albert Broscheit

**Affiliations:** Department of Anesthesiology and Critical Care, University of Wuerzburg, Oberduerrbacher Str.6, 97080 Wuerzburg, Germany

**Keywords:** APC, Ischemia–reperfusion injury, Mitochondria, Apoptosis

## Abstract

Volatile anesthetic-induced preconditioning (APC) has shown to have cardiac and cerebral protective properties in both pre-clinical models and clinical trials. Interestingly, accumulating evidences demonstrate that, except from some specific characters, the underlying molecular mechanisms of APC-induced protective effects in myocytes and neurons are very similar; they share several major intracellular signaling pathways, including mediating mitochondrial function, release of inflammatory cytokines and cell apoptosis. Among all the experimental results, cortical spreading depolarization is a relative newly discovered cellular mechanism of APC, which, however, just exists in central nervous system. Applying volatile anesthetic preconditioning to clinical practice seems to be a promising cardio-and neuroprotective strategy. In this review, we also summarized and discussed the results of recent clinical research of APC. Despite all the positive experimental evidences, large-scale, long-term, more precisely controlled clinical trials focusing on the perioperative use of volatile anesthetics for organ protection are still needed.

## Background

Perioperative organ protection has always been a critical issue for both anesthesiologists and surgeons. Neurological and cardiac outcome severely affects postoperative morbidity and mortality. With the development of the modern medicine, the population of aging patients with ischemic heart and brain diseases has significantly increased. A large number of patients, who have already suffered heart attack or cerebral stoke, have to risk the ischemia–reperfusion (I/R) damage again in surgical operations, the results could be devastating.

**Fig. 1:**
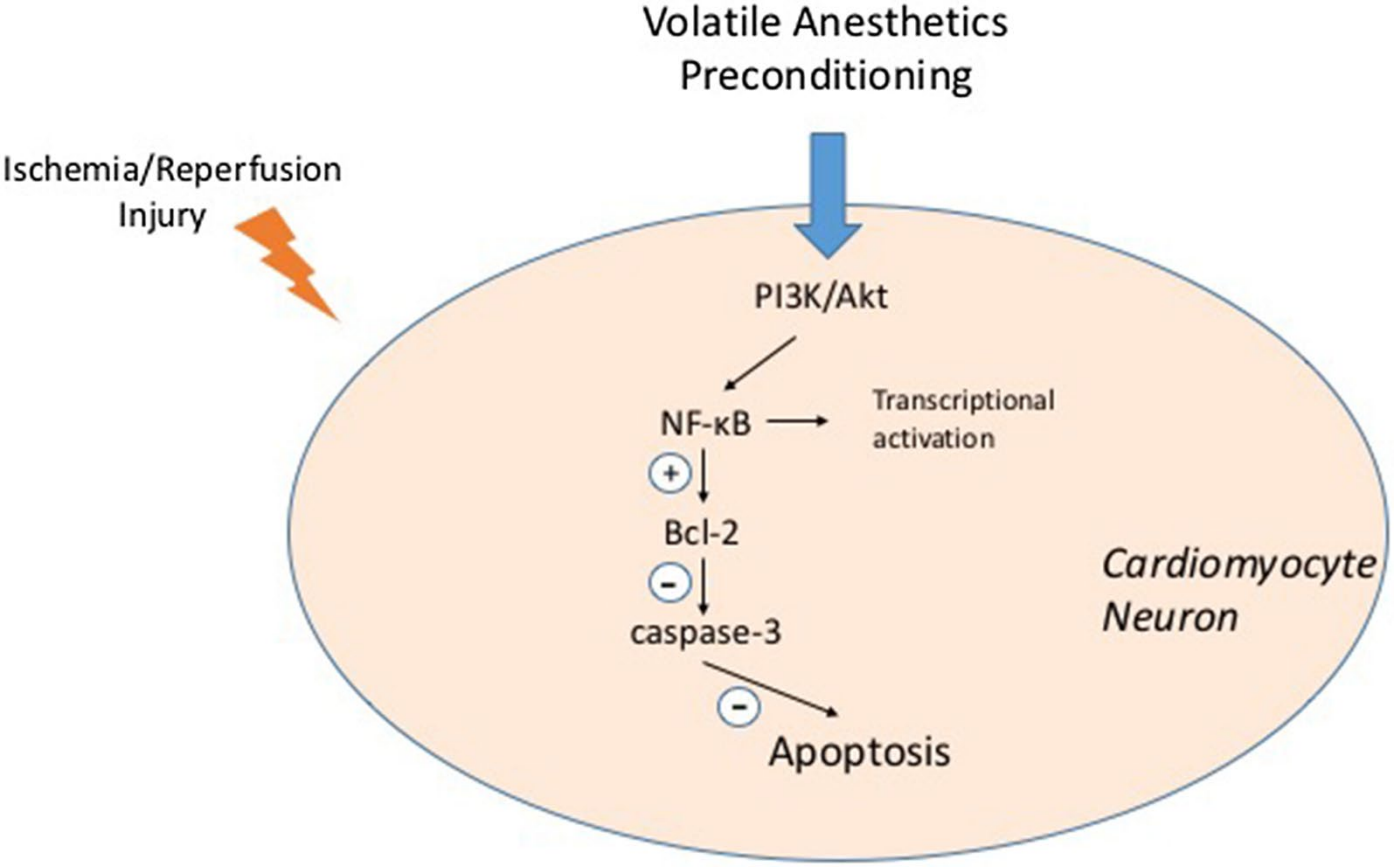
Similar molecular signaling pathways of anti-apoptotic effect in cardiomyocytes and neurons induced by APC

Cardiomyocytes and neurons are both highly hypoxia-sensitive cells with, however, very different properties. Myocytes are muscle cells and responsible for the systolic and diastolic functions. They are regulated mainly by beta adrenergic system. Neurons, on the contrary, are electrically excitable cells. They connect to each other and transfer electrical and chemical signals via synapse. The two systems are separated from each other by blood–brain-barrier, which has highly selective permeability. Interestingly, the ischemic conditioning phenomenon has been demonstrated in both organs. Volatile anesthetic (VA)-induced preconditioning contributes to both cardiac and cerebral protection in the I/R injury [1, 2]. Sevoflurane-, isoflurane- and desflurane-induced preconditioning have been confirmed to be capable of reducing the infarct size, improving perioperative cardiac function significantly [3, 4]. In neurological experiments, these medical gases also exert their remarkable protective properties against cerebral ischemic injury [5]. How could APC achieve ‘multiorgan’ protection? A large number of studies have been applied to understand this essential phenomenon. The aim of this review is to provide an overview and compare the molecular actions and subsequent cellular signaling cascade mediated by APC in these two totally different systems.

## Mitochondrial function

### mK_ATP_ channels

In cardiomyocytes and neurons, mitochondria produce the majority of cellular adenosine triphosphate (ATP) and pathological reactive oxygen species (ROS) during I/R injury [6, 7]. They also play a key role in VA-induced preconditioning. Numerous studies have demonstrated that sevoflurane and other VAs protect the myocardium and brain during I/R injury via the mitochondrial ATP-sensitive potassium (mK_ATP_) channels, opening of mK_ATP_ channels results in potassium influx, slowing of calcium overload in the mitochondria, the production of reactive oxygen species and the activation of multiple downstream kinases and molecular cascades of cardiac protection, and this has been considered as a critical step in APC [8, 9]. It is verified that isoflurane can directly activate the human cardiac mK_ATP_ channels in vitro. ATP-sensitive K^+^ currents were significantly increased after isoflurane exposure, and this effect was completely abolished by mK_ATP_ channels blocker 5-hydroxydecanoate (5-HD) [10]. Riess et al. provided the first evidence that sevoflurane is able to prevent mitochondrial matrix volume (MMV) contraction during ischemia, and this effect is mediated via mK_ATP_ channel opening [11]. Among all the signaling kinases which are involved in cardiovascular functions, protein kinase C (PKC) and its subgroup PKCε are believed to be key signaling pathway associated with mK_ATP_ channel-mediated cardiac protection [8, 12, 13]. Wang et al. demonstrated that sevoflurane preconditioning exhibits a delayed cardioprotection against I/R injury by increasing PKCε phosphorylation, and this effect is inhibited by mK_ATP_ channel blocker 5-HD [14]. Similar effects were also reported by Kaneda et al. [15] and Weber et al. [16] confirming the key role of PKCε/mK_ATP_ signaling pathway in cardiac protective effect induced by APC.

Mitochondrial K_ATP_ channel proteins, which are partially purified from rat brain mitochondria, exhibit ligand-binding properties similar to those of heart mK_ATP_ channels, and the amount of mK_ATP_ channels in brain seems to be much higher than in the heart, suggesting the crucial role of mK_ATP_ channel in central nervous system [17]. Numerous studies have proved that VA preconditioning provides neuroprotection via mK_ATP_ channel both in vivo and in vitro. Kehl et al. demonstrated that the preconditioning induced by sevoflurane was abolished by 5HD, a mK_ATP_ channel blocker, in rat hippocampal slices [18]. Likewise, it was observed that the opening of mK_ATP_ channel mimicked delayed preconditioning induced by sevoflurane, whereas sevoflurane postconditioning was also blocked by 5-HD given at the end of ischemia [19]. Interestingly, it was also reported for the first time by the same research team [19] that neuroprotective effects mediated by sevoflurane were observed when sevoflurane was given at the onset of reperfusion, and this effect was lost when it is given 5 min after the onset of reperfusion, indicating the time, duration and other factors also associate and play potential relevance in APC. Moreover, it is found recently that the P38 phosphorylation was decreased after the administration of 5-HD, suggesting that mK_ATP_ channel opening and p38 phosphorylation are both involved sevoflurane-induced preconditioning and p38 MAPK activation may be a downstream of opening mK_ATP_ channels [20]. Similar to cardioprotection, PKCε is also involved in APC-mediated neuroprotection. Ye et al. demonstrated that application of 5-HD 30 min before sevoflurane exposure could not only attenuate its beneficial effects in reducing neurological deficit scores and brain infarct volume, but also inhibit the translocation of PKC ε to the membrane fraction at 24 h after reperfusion [21]. This result also indicates the role of PKC ε as the upstream target of mK_ATP_ channels and thus the similar cell signal pathway in APC-mediated protective effects in both cardiac and neural system.

### mPTP

The mitochondrial permeability transition with the formation of mitochondrial permeability transition pore (mPTP) has been identified as a key mediator in the process of mitochondrial dysfunction. The formation of mPTP could be induced under the pathological conditions, such as Ca^2+^ overload and oxidative stress due to ischemia or chronic heart failure, and the opening of mPTP causes mitochondrial dysfunction with lipid peroxidation, ATP hydrolysis, matrix swelling and ultimately cell death [22, 23]. Moreover, using pharmacological inhibitor of mPTP cyclosporine A (CsA) demonstrated protective effects against myocardial ischemic–reperfusion injury through reducing the infarct size and apoptotic cardiomyocyte death [24]. Accumulating data have shown that inhibition opening of mPTP is related to volatile anesthetic-induced preconditioning: Pravdic et al. reported that isoflurane preconditioning delays mPTP opening under conditions of oxidative stress in rat cardiomyocytes [25]; similar effect of isoflurane preconditioning was confirmed again in a recent study by Sepac et al. with cardiomyocytes derived from human embryonic stem cells (hESC), mPTP opening time of cardiomyocytes was significantly delayed after 15-min pretreatment with 0.5 mM isoflurane [26]; Onishi et al. demonstrated the direct evidence that sevoflurane preconditioning, similar to CsA, inhibits calcium-induced mPTP opening by increasing the threshold, and this cardioprotective effect is mediated by inhibition of glycogen synthase kinase 3ß (GSK-3β) by phosphorylation through phosphatidylinositide 3-kinases/protein kinase B (PI3K/AKt) pathway [27].

Mitochondrial dysfunction contributes to neurologic disorders, including neurodegenerative diseases and stroke, where, similar to cardiac I/R injury, excessive Ca^2+^ uptake can activate the opening of mPTP, which causes uncoupled ATP synthesis, following with neuronal death [28, 29]. Recent studies confirmed that inhibition of mPTP opening is also one of the most important cellular mechanisms of APC-induced neuroprotection. It is demonstrated by Ye’s group that sevoflurane preconditioning protects mitochondria from cerebral I/R injury by hyperpolarizing mitochondrial membrane potential in ischemic brain issue and inhibiting Ca^2+^-induced mitochondrial permeability transition. Ischemic rats pretreated with sevoflurane exhibited a significantly diminished neurological deficit, and the infarct volume was reduced by 34% [30].

### ROS

Reactive oxygen species were known to be released under the oxidative stress. Overproduction of ROS can result in cellular dysfunction and has a key role in various chronic diseases such as cardiac vascular diseases and neurodegenerative diseases. In I/R injury, large amounts of ROS are released during reperfusion, produce oxidative damage to lipids, proteins, nucleic acids and finally severe tissue damage [7, 31, 32]. Recent evidence suggests that, in contrast, small amounts of ROS released from mitochondria could induce preconditioning and contribute to cardioprotection; this effect could be abolished with ROS scavengers [33, 34]. VAs trigger the production of a small amount of ROS, which appear to be critical events at the onset of cell signaling cascade of preconditioning against myocardial injury [6, 33, 35]. Pretreatment with sevoflurane at 2% or desflurane at 7% before a 30-min hypoxic period enhanced the recovery of myocardial contraction of human atrial trabeculae; however, this function was inhibited by *N*-[2-mercaptopropionyl]-glycine (MPG), a ROS scavenger, indicating the role of ROS in sevoflurane- and desflurane-induced preconditioning against cardiac I/R injury [36]. Isoflurane was demonstrated to have the ability to enhance the production of ROS in hESC-derived cardiomyocytes [26]. The in vitro study of Sedlic et al. showed that the rate of ROS generation was significantly increased during the application of desflurane or sevoflurane, and after anesthetic washout, the rate of ROS production was decreased [37]. The ROS production elicited by VAs might mediate mitochondrial uncoupling in APC signaling cascade.

In central nervous system, ROS was also demonstrated to be involved in VA-preconditioning-induced neuroprotection. ROS generation and its role in triggering of APC was examined by Velly et al. on mixed cortical neuronal–glial cell cultures subjected to transient oxygen–glucose deprivation (OGD) [38]. The result showed that sevoflurane exposure during preconditioning induced a significant increase in ROS levels, which was prevented by ROS scavengers. In a rabbit spinal cord ischemic injury model, isoflurane demonstrated a delayed neuroprotective effect via the release of free radicals [39]. It has also been proved in vivo with a rat middle cerebral artery occlusion (MCAO) model in a recent study, that the initial oxidative stress generated by sevoflurane preconditioning may trigger cascades that finally lead to ischemic tolerance [40]. However, although transient production of ROS is necessary for APC, chronic oxidative stress still appears to result in mitochondrial damage and cell death, thus decrease cardioprotection or neuroprotection after APC [41, 42].

## Inflammatory cytokines

Oxidative stress and inflammatory reactions contribute to I/R injury. In the ischemic phase, the balance of endogenous oxidants and antioxidants is disrupted, and pro-inflammatory cytokines like TNF-α, IL-1, and reactive oxygen species are largely produced in reperfusion phase. TNF-α and other pro-inflammatory cytokines cause further oxidative stress through inducing phosphorylation and the activation of nuclear factor κB (NF-κB). As a transcription factor, NF-κB is widely involved and regulates the expression of a large number of cytokines, chemokines and pro-inflammatory enzymes, such as TNF-α, IL1, IL8, iNOS, cyclooxygenase-2 and adhesion molecules, which are critical to generate inflammation and apoptosis [43, 44].

Volatile anesthetic-induced preconditioning has been reported to protect myocardium against I/R injury by attenuating the activation of NF-κB and thus subsequently suppress the expression of NF-κB-dependent inflammatory cytokines [45–47]. Wang et al. demonstrated in an in vivo coronary artery occlusion experiment that sevoflurane preconditioning attenuates the upregulation of NF-κB with its two subunits p50 and p65, and subsequently decreases the expression of inflammatory proteins TNF-α and intercellular adhesion molecule-1 (ICAM-1). Administration of the NF-κB inhibitor parthenolide (PTN) before or after exposure to sevoflurane abolished the benefit effect as reducing the infarct sizes [48]. In another research study, using electrophoretic mobility shift assay, NF-κB activity was determined. After preconditioning with sevoflurane, NF-κB binding activity and activation were significantly downregulated, with reduced expression of TNF-α, ICAM-1, IL-1 and iNOS during myocardial I/R injury [44].

In central nervous system, pathological conditions cause the overactivation of microglia, and a large quantity of pro-inflammatory cytokines can be subsequently released from activated microglia [49, 50]. Toll-like receptor 4 (TLR4) is expressed primarily in microglia and plays a key role in the activation of microglia and the production of inflammatory cytokines in central nervous system [51–53]. NF-κB has been confirmed as a downstream factor of TLR4 signaling pathway [54, 55], and similar to the research results in cardiovascular system, after activation, NF-ΚB moves to the nucleus, and triggers the transcription of genes responsible for inflammatory reactions and eventually lead to neuron damage. Moreover, TLR4/NF-κB signaling pathway also mediates the neuroprotective effect of APC. Sun et al. demonstrated in an in vitro OGD experiment with primary neurons/astrocytes culture that 2% isoflurane preconditioning has similar effects as the TLR4-specific inhibitor CLI-095: downregulated the expression of TLR4, IL-1ß, TNF-α, and up-regulated NF-κB inhibitor IκB-α [56]. In vivo study of the same research group showed that isoflurane preconditioning attenuated neurological deficits, infarct volume against ischemic stroke, and inhibited microglial activation. Molecular tests also showed the same results as the in vitro study. There are also research reports demonstrating that sevoflurane provides direct neuroprotective effects in suppressing the activation of NF-kappa B and expression of inflammatory cytokines against focal ischemic brain injury [57, 58].

## Apoptosis

Ischemia causes the destruction of cardiomyocytes and leads to necrosis. However, the infarct size of myocardium is determined not only by the necrosis after ischemia, but also the apoptotic cell death triggered and principally developed during the phase of reperfusion. Apoptosis is associated with the extension of infarction over the time of prolonged reperfusion, and has been considered as the predominant form of both myocardial and cerebral ischemia- and reperfusion-related cell death. The importance of apoptosis in cell death following reperfusion has been demonstrated in vivo; the results show that the level of apoptosis is dependent on the duration of reperfusion. Apoptosis can be triggered by ischemia but reperfusion accelerates the process [59, 60].

Several protein families are involved in apoptosis, including B cell lymphoma-2 (Bcl-2), Bax, Bak and caspases. Bcl-2 family is one of the most important regulatory factors in cell apoptosis. Because of its anti-apoptotic effect, the role of Bcl-2 family in APC-induced cardio- and neuroprotection has also been widely investigated [61, 62]. In myocardial I/R injury model, VAs were found to have anti-apoptotic effects as well as reduce myocardial infarct size. Raphael et al. demonstrated that isoflurane preconditioning attenuates infarct size and myocardial apoptosis after I/R, via PI3K/Akt signaling and modulation of anti-apoptotic Bcl-2 family proteins [63]. Interestingly, evidence shows that, unlikely to the role of triggering the production of inflammatory cytokines, the activation of NF-kappa B is involved in upregulating anti-apoptotic protein Bcl-2 resulting in significant decrease in cell apoptosis and mediates cytoprotective effects [64, 65]. Furthermore, this mechanism was also consistent with the experimental results reported by Wang et al. in a study of APC with sevoflurane [48]. Sevoflurane-produced myocardial protection during preconditioning was through upregulation of anti-apoptotic protein Bcl-2, and ultimately decreasing caspase-3 expression and apoptosis. This benefit effect was abolished by NF-kappa B inhibitor PTN.

Likewise, anti-apoptotic effect of APC was confirmed with neurons. Preconditioning with 2% sevoflurane or 1.5% isoflurane for 60 min provided neuroprotection by upregulation of anti-apoptotic genes (Aven, Bcl-2, Bcl2l2, and Prok2) and downregulation of pro-apoptotic genes (Tnf, Tnfrsf10b and Tp53) [66]. A very recent in vivo study proved that APC with sevoflurane reduced apoptosis in rat brains, which was also associated with increased ratios of anti-apoptotic Bcl-2 family proteins, with decreased activation of JNK and p53 pathways, and finally decreased caspase-3 expression [67]. Zhao et al. reported that isoflurane preconditioning at a clinically relevant concentration improved long-term neurological outcome in neonatal rats under hypoxic ischemic brain injury. This effect involved an increased expression of anti-apoptotic protein Bcl-2 [68]. Sevoflurane preconditioning has been proved to show neuroprotective effects in terms of improving neurofunctional outcome and the attenuating infarct volume significantly. The anti-apoptotic effect was observed up to 7 days after cerebral I/R injury [69]. PI3K/Akt signaling pathway and its downstream target GSK-3ß were implicated to be involved in caspase-dependent mechanisms of cell apoptosis [70]. Ischemia injury induces Akt phosphorylation at Ser473, sevoflurane preconditioning inhibited the hyperphosphorylation in a MCAO model, which thereby phosphorylated/inactivated GSK-3ß and represented a neuroprotective effect [71] (Fig. 1).

## Cortical spreading depolarization

Cortical spreading depolarization (CSD) is a relative newly discovered mechanism for secondary ischemic neurological damage. It appears that spreading depolarization does not have a physiological role in normal tissue but can be instigated under the pathological conditions, such as mechanical damages, noxious chemical agents, ischemia and propagates slowly across the cerebral cortex [72, 73]. In the last decade, more and more clinical studies showed that CSDs are correlated to a subsequent brain tissue injury, neurological deficits and worse patient outcome [74–76]. Takagaki et al. examined and compared the effects of isoflurane and propofol on CSDs in a rodent MCAO model and found out that isoflurane reduced the occurrence of CSDs significantly in comparison to propofol. Furthermore, cerebral blood flow and plasma glucose are significantly lower under the propofol suggesting a worse oxygen and glucose supply in propofol group [77]. The hypothesis that VAs contribute to neuroprotection is further validated by the research group of Kudo et al. [78]. Isoflurane achieved significant suppression of CSD frequency and CSD propagation speed.

## Clinical aspects

Accumulating clinical studies have confirmed the benefits of intraoperative administration of VAs in patients undergoing coronary artery bypass surgery [79–81]. A meta-analysis of randomized clinical trials involving 1922 patients undergoing cardiac surgery showed that, in comparison with total intravenous anesthesia (TIVA), desflurane and sevoflurane achieved significant reductions of myocardial infarctions [2.4% in the VA group vs 5.1% in the TIVA group, odds ratio (OR) 0.51] and all-cause mortality (0.4% vs 1.6%, OR 0.31). Besides these benefit effects, the use of VAs was also associated with a shorter duration of intensive care unit stay and need for mechanical ventilation [82]. Isoflurane was reported to reduce troponin release in a randomized clinical trial involving forty-five patients undergoing elective off-pump coronary artery bypass surgery [83]. Guerrero Orriach and colleagues found out that the use of sevoflurane attenuated significantly the postoperative levels of troponin I at 24 h and N-terminal pro-brain natriuretic peptide (NT–proBNP) at 24 and 48 h in coronary surgery patients. Furthermore, the use of inotropic drugs was also significantly reduced at 24 and 48 h postoperatively [84]. Amr et al. demonstrated in a recent clinical trial with 45 patients undergoing coronary artery bypass graft surgery that preconditioning with isoflurane 2.5% improved significantly perioperative hemodynamic function, and reduced postoperative level of cardiac troponin I and creatine kinase isoenzyme MB (CK-MB) release [85].

Based on experimental studies, VAs have shown promising results against cerebral ischemic injury. However, similar results in human trials are much less certain, clinic evidence is still very limited [86, 87]. Because of their vasodilative effect, VAs could lead to higher intracranial pressure; therefore, propofol still remains as the first choice of anesthetic for severe head trauma (SHT) or patients with elevated intracranial pressure (ICP) [88]. But there are also studies demonstrating that sevoflurane has nearly no impact on cerebrovascular autoregulation and the ICP below 1.0 minimum alveolar concentration (MAC). These results make sevoflurane a possible choice for neurological surgeries [89, 90]. Schoen et al. reported that sevoflurane improved short-term postoperative cognitive function in patients undergoing on-pump cardiac surgery compared with propofol [91]. Dabrowski et al. confirmed that [92] sevoflurane and isoflurane attenuated plasma matrix metalloproteinase-9 (MMP-9), glial fibrillary acidic protein (GFAP) concentrations, which are considered to be the specific biochemical markers of brain injury [93, 94], and brain magnesium disorders in patients undergoing coronary artery bypass graft surgery. In addition, isoflurane was also reported capable of providing beneficial effects on regional cerebral blood flow in patients with subarachnoid hemorrhage, without any relevant elevation of ICP [95]. Another very recent clinical study from neonatal intensive care unit (NICU) showed that, in patients with low ICP or only moderately elevated values, isoflurane was capable of reducing the in cerebral oxygen extraction without significant intracranial hypertension [96], indicating a promising strategy of neuroprotection for patents in ICU or NICU. All these clinical research results suggest a promising prospect of VAs in cerebral protection for patients undergoing neurosurgical or non-neurosurgical procedures. However, there are also clinical studies with confusing results on VAs *vs* propofol in neurological surgeries. In a recent systemic clinic review and meta-analysis of Chui et al., VAs and propofol were compared for maintenance of anesthesia during craniotomy operations. Propofol and VAs did not show any significant differences in brain relaxation scores, but propofol-anesthesia maintained lower ICP and higher cerebral perfusion pressure (CPP) values. Postoperative complications were similar between two groups; however, postoperative nausea and vomiting were more associated with VA anesthesia [97]. The similar conflicting evidences have also been reported in other clinical studies [5, 98] (Table 1).

**Table 1:** Clinical studies of the protective effects of VAs

	Surgery	Patients and number (*n*)	Volatile anesthetics and dose	Outcome	Refs.
Cardioprotection	Cardiopulmonary bypass	*n* = 200	Sevoflurane 0.5–2%	Increase of troponin I ↓ Cardiac output ↑ Inotropic support ↓ Duration of stay in the ICU and hospital ↓	De Hert et al. [79]
	Cardiopulmonary bypass	Elderly high-risk^a^ patients, *n* = 45	Sevoflurane 0.5–2% Desflurane 1–4%	Decrease in cardiac index (CI) post-CPB ↓ Inotropic support ↓	De Hert et al. [80]
	Off-pump coronary artery bypass grafting	*n* = 48	Sevoflurane 1.0 MAC and 1.5 MAC	Post-surgical cardiac Troponin I ↓	Wang et al. [81]
	Off-pump coronary artery bypass	*n* = 45	Isoflurane 1.0–2.5%	Post-surgical cardiac index ↑ Increase of troponin T ↓	Tempe et al. [83]
	Off-pump coronary artery bypass	*n* = 60	Sevoflurane 0.7 and 1 MAC	Increase of troponin I ↓ Postoperative NT-proBNP^b^ ↓ Inotropic support ↓	Guerrero Orriach et al. [84]
	On-pump coronary artery bypass	*n* = 45	Isoflurane 2.5% 10 min^c^	Hemodynamic recovery ↑ Cardiac troponin T ↓ CK-MB release ↓ Inotropic support ↓	Amr et al. [85]
Cerebral protection	Cardiopulmonary bypass	*n* = 128	Sevoflurane 0.6–1 MAC	Cognitive function ↑	Schoen et al. [91]
	Cardiopulmonary bypass	*n* = 92	Sevoflurane 0.5 MAC Isoflurane 0.5 MAC	Postoperative Mg disorders ↓ Increase in plasma MMP-9 and GFAP^d^ concentration ↓	Dabrowski et al. [92]

*CPB* cardiopulmonary bypass, *MAC* minimal alveolar concentration

^a^Elderly high-risk: older than 70 years with three-vessel disease and an ejection fraction less than 50% with impaired length-dependent regulation of myocardial function

^b^N-terminal pro-brain natriuretic peptide

^c^Preconditioning with a 10-min exposure to isoflurane 2.5% followed by 5-min washout

^d^Sensitive marker of brain injury: matrix metalloproteinase-9 (MMP-9), glial fibrillary acidic protein (GFAP)

Not only in cerebral- and cardiac systems, anti-apoptotic effects of volatile anesthetic were also reported in hepatic ischemia–reperfusion injury [99, 100]. Treatment with sevoflurane significantly improved the pulmonary function of lung grafts by reducing levels of pro-inflammatory cytokines [101]. Desflurane preconditioning demonstrated significant renal protective effects in a rabbit model of acute I/R injury [102]. In a recent study with human volunteers, Lucchinetti et al. reported the protective effect of sevoflurane in human endothelium though the inhibition of leukocyte adhesion [103]. In another randomized clinical trial with seventy-two patients undergoing coronary artery bypass graft surgery, preconditioning by sevoflurane reduced the transcript levels for platelet–endothelial cell adhesion molecule-1 (PECAM-1), indicating the protective effect on endothelial system, as well as attenuated significantly the incidence of late cardiac events during the first year after surgery comparing with the placebo group [104]. It is well known that vascular endothelial function is critically involved in a broad range of diseases as well as ischemic–reperfusion injury [105–107]. Endothelial stability plays a key role in maintaining physical vascular function and coagulation status. Hence, VA preconditioning could be served as a ubiquitous ‘multiorgan’ protection through reducing endothelial dysfunction in I/R injury.

## Conclusions

Extensive evidences from animal experimental studies have proved that VAs have beneficial effect to protect the myocytes and neurons from ischemia/reperfusion injury. The protective cellular signaling pathways are extremely similar in both cardiovascular and central nervous systems. There are also promising results from clinical trials with beneficial effects of APC for the patients undergoing cardiac and neurological surgeries. Some research results still remain confusing and conflicting, which could be, however, the results of different timing and patterns of administration, or inappropriate doses. Further work including more randomized and qualitative clinical trials is still required. Determining the time effect, the concentration of VAs during operations, and long-term clinic outcome should be focused to clarify and guarantee the best protective effects of VAs in clinical practice.

## Abbreviations

APC: volatile anesthetic-induced preconditioning
VAs: volatile anesthetics
I/R: ischemia–reperfusion
ROS: reactive oxygen species
ATP: adenosine triphosphate
mK_ATP_: mitochondrial ATP-sensitive potassium
5-HD: 5-hydroxydecanoate
MMV: mitochondrial matrix volume
PKC: protein kinase C
mPTP: mitochondrial permeability transition pore
CsA: cyclosporine A
hESC: human embryonic stem cells
GSK-3β: glycogen synthase kinase 3ß
PI3K: phosphatidylinositide 3-kinases
AKt: protein kinase B
MPG: *N*-[2-mercaptopropionyl]-glycine
OGD: oxygen glucose deprivation
MCAO: middle cerebral artery occlusion
NF-κB: nuclear factor κB
ICAM-1: intercellular adhesion molecule-1
PTN: parthenolide
TLR4: Toll-like receptor 4
Bcl-2: B cell lymphoma-2
CSD: cortical spreading depolarizations
TIVA: total intravenous anesthesia
OR: odds ratio
NT-proBNP: N-terminal pro-brain natriuretic peptide
CK-MB: creatine kinase isoenzyme MB
ICP: intracranial pressure
MAC: minimum alveolar concentration
MMP-9: matrix metalloproteinase-9
GFAP: glial fibrillary acidic protein
NICU: neonatal intensive care unit
PECAM-1: platelet–endothelial cell adhesion molecule-1

## References

[1] Werner C, Möllenberg O, Kochs E (1995). Schulte J am Esch. Sevoflurane improves neurological outcome after incomplete cerebral ischaemia in rats. Br J Anaesth.

[2] Engelhard K, Werner C, Reeker W, Lu H, Möllenberg O, Mielke L, Kochs E (1999). Desflurane and isoflurane improve neurological outcome after incomplete cerebral ischaemia in rats. Br J Anaesth.

[3] Lutz M, Liu H (2006). Inhaled sevoflurane produces better delayed myocardial protection at 48 versus 24 hours after exposure. Anesth Analg.

[4] Chiari PC, Pagel PS, Tanaka K, Krolikowski JG, Ludwig LM, Trillo RA, Puri N, Kersten JR, Warltier DC (2004). Intravenous emulsified halogenated anesthetics produce acute and delayed preconditioning against myocardial infarction in rabbits. Anesthesiology.

[5] Deng J, Lei C, Chen Y, Fang Z, Yang Q, Zhang H, Cai M, Shi L, Dong H, Xiong L (2014). Neuroprotective gases–fantasy or reality for clinical use?. Prog Neurobiol.

[6] Agarwal B, Stowe DF, Dash RK, Bosnjak ZJ, Camara AK (2014). Mitochondrial targets for volatile anesthetics against cardiac ischemia-reperfusion injury. Front Physiol.

[7] Christophe M, Nicolas S (2006). Mitochondria: a target for neuroprotective interventions in cerebral ischemia-reperfusion. Curr Pharm Des.

[8] Swyers T, Redford D, Larson DF (2014). Volatile anesthetic-induced preconditioning. Perfusion.

[9] Garlid KD, Dos Santos P, Xie ZJ, Costa AD, Paucek P (2003). Mitochondrial potassium transport: the role of the mitochondrial ATP-sensitive K(+) channels in cardiac function and cardioprotection. Biochim Biophys Acta.

[10] Jiang MT, Nakae Y, Ljubkovic M, Kwok WM, Stowe DF, Bosnjak ZJ (2007). Isoflurane activates human cardiac mitochondrial adenosine triphosphate-sensitive K+ channels reconstituted in lipid bilayers. Anesth Analg.

[11] Riess ML, Costa AD, Carlson R, Garlid KD, Heinen A, Stowe DF (2008). Differential increase of mitochondrial matrix volume by sevoflurane in isolated cardiac mitochondria. Anesth Analg.

[12] Churchill EN, Mochly-Rosen D (2007). The roles of PKCdelta and epsilon isoenzymes in the regulation of myocardial ischaemia/reperfusion injury. Biochem Soc Trans.

[13] Li H, Lang XE (2015). Protein kinase C signaling pathway involvement in cardioprotection during isoflurane pretreatment. Mol Med Rep.

[14] Wang C, Hu SM, Xie H, Qiao SG, Liu H, Liu CF (2015). Role of mitochondrial ATP-sensitive potassium channels-mediated PKC-ε in delayed protection against myocardial ischemia/reperfusion injury in isolated hearts of sevoflurane-preconditioned rats. Br J Med Biol Res.

[15] Kaneda K, Miyamae M, Sugioka S, Okusa C, Inamura Y, Domae N, Kotani J, Figueredo VM (2008). Sevoflurane enhances ethanol-induced cardiac preconditioning through modulation of protein kinase C, mitochondrial KATP channels, and nitric oxide synthase, in guinea pig hearts. Anesth Analg.

[16] Weber NC, Toma O, Damla H, Wolter JI, Schlack W, Preckel B (2006). Upstream signaling of protein kinase C-epsilon in xenon-induced pharmacological preconditioning. Implication of mitochondrial adenosine triphosphate dependent potassium channels and phosphatidylinositol-dependent kinase-1. Eur J Pharmacol.

[17] Bajgar R, Seetharaman S, Kowaltowski AJ, Garlid KD, Paucek P (2001). Identification and properties of a novel intracellular (mitochondrial) ATP-sensitive potassium channels in brain. J Biol Chem.

[18] Kehl F, Payne RS, Roewer N, Schurr A (2004). Sevoflurane-induced preconditioning of rat brain in vitro and the role of KATP channels. Brain Res.

[19] Adamczyk S, Robin E, Simerabet M, Kipnis E, Tavernier B, Vallet B, Bordet R, Lebuffe G (2010). Sevoflurane pre- and post-conditioning protect the brain via the mitochondrial K ATP channels. Br J Anaesth.

[20] Ye Z, Guo Q, Wang N, Xia P, Yuan Y, Wang E (2012). Delayed neuroprotection induced by sevoflurane via opening mitochondrial ATP-sensitive potassium channels and p38 MAPK phosphorylation. Neurol Sci.

[21] Ye Z, Huang YM, Wang E, Zuo ZY, Guo QL (2012). Sevoflurane-induced delayed neuroprotection involves mitoK(ATP) channels opening and PKC ε activation. Mol Biol Rep.

[22] Bernardi P, Di Lisa F (2015). The mitochondrial permeability transition pore: molecular nature and role as a target in cardioprotection. J Mol Cell Cardiol.

[23] Lotz C, Zhang J, Fang C, Liem D, Ping P (2015). Isoflurane protects the myocardium against ischemic injury via the preservation of mitochondrial respiration and its supramolecular organization. Anesth Analg.

[24] Argaud L, Gateau-Roesch O, Muntean D, Chalabreysse L, Loufouat J, Robert D, Ovize M (2005). Specific inhibition of the mitochondrial permeability transition prevents lethal reperfusion injury. J Mol Cell Cardiol.

[25] Pravdic D, Sedlic F, Mio Y, Vladic N, Bienengraeber M, Bosnjak ZJ (2009). Anesthetic-induced preconditioning delays opening of mitochondrial permeability transition pore via protein Kinase C-epsilon-mediated pathway. Anesthesiology.

[26] Sepac A, Sedlic F, Si-Tayeb K, Lough J, Duncan SA, Bienengraeber M, Park F, Kim J, Bosnjak ZJ (2010). Isoflurane preconditioning elicits competent endogenous mechanisms of protection from oxidative stress in cardiomyocytes derived from human embryonic stem cells. Anesthesiology.

[27] Onishi A, Miyamae M, Kaneda K, Kotani J, Figueredo VM (2012). Direct evidence for inhibition of mitochondrial permeability transition pore opening by sevoflurane preconditioning in cardiomyocytes: comparison with cyclosporine A. Eur J Pharmacol.

[28] Sims NR, Muyderman H (2010). Mitochondria, oxidative metabolism and cell death in stroke. Biochim Biophys Acta.

[29] Perez-Pinzon MA, Stetler RA, Fiskum G (2012). Novel mitochondrial targets for neuroprotection. J Cereb Blood Flow Metab.

[30] Ye R, Yang Q, Kong X, Li N, Zhang Y, Han J, Xiong L, Liu X, Zhao G (2012). Sevoflurane preconditioning improves mitochondrial function and long-term neurologic sequelae after transient cerebral ischemia: role of mitochondrial permeability transition. Crit Care Med.

[31] Sanders LH, Greenamyre JT (2013). Oxidative damage to macromolecules in human Parkinson disease and the rotenone model. Free Radic Biol Med.

[32] Sugamura K, Keaney JF (2011). Reactive oxygen species in cardiovascular disease. Free Radic Biol Med.

[33] Tanaka K, Weihrauch D, Kehl F, Ludwig LM, LaDisa JF, Kersten JR, Pagel PS, Warltier DC (2002). Mechanism of preconditioning by isoflurane in rabbits: a direct role for reactive oxygen species. Anesthesiology.

[34] Vanden Hoek TL, Becker LB, Shao Z, Li C, Schumacker PT (1998). Reactive oxygen species released from mitochondria during brief hypoxia induce preconditioning in cardiomyocytes. J Biol Chem.

[35] Mullenheim J, Ebel D, Frassdorf J, Preckel B, Thanmer V, Schlack W (2002). Isoflurane preconditions myocardium against infarction via release of free radicals. Anesthesiology.

[36] Hanouz JL, Zhu L, Lemoine S, Durand C, Lepage O, Massetti M, Khayat A, Plaud B, Gérard JL (2007). Reactive oxygen species mediate sevoflurane- and desflurane-induced preconditioning in isolated human right atria in vitro. Anesth Analg.

[37] Sedlic F, Pravdic D, Ljubkovic M, Marinovic J, Stadnicka A, Bosnjak ZJ (2009). Differences in production of reactive oxygen species and mitochondrial uncoupling as events in the preconditioning signaling cascade between desflurane and sevoflurane. Anesth Analg.

[38] Velly LJ, Canas PT, Guillet BA, Labrande CN, Masmejean FM, Nieoullon AL, Gouin FM, Bruder NJ, Pisano PS (2009). Early anesthetic preconditioning in mixed cortical neuronal-glial cell cultures subjected to oxygen-glucose deprivation: the role of adenosine triphosphate dependent potassium channels and reactive oxygen species in sevoflurane-induced neuroprotection. Anesth Analg.

[39] Sang H, Cao L, Qiu P, Xiong L, Wang R, Yan G (2006). Isoflurane produces delayed preconditioning against spinal cord ischemic injury via release of free radicals in rabbits. Anesthesiology.

[40] Yang Q, Dong H, Deng J, Wang Q, Ye R, Li X, Hu S, Dong H, Xiong L (2011). Sevoflurane preconditioning induces neuroprotection through reactive oxygen species-mediated up-regulation of antioxidant enzymes in rats. Anesth Analg.

[41] Nguyen LT, Rebecchi MJ, Moore LC, Glass PS, Brink PR, Liu L (2008). Attenuation of isoflurane-induced preconditioning and reactive oxygen species production in the senescent rat heart. Anesth Analg.

[42] Sedlic F, Sepac A, Pravdic D, Camara AK, Bienengraeber M, Brzezinska AK, Wakatsuki T, Bosnjak ZJ (2010). Mitochondrial depolarization underlies delay in permeability transition by preconditioning with isoflurane: roles of ROS and Ca2+. Am J Physiol Cell Physiol.

[43] Valen G, Yan ZQ, Hansson GK (2001). Nuclear factor kappa-B and the heart. J Am Coll Cardiol.

[44] Zhong C, Zhou Y, Liu H (2004). Nuclear factor kappaB and anesthetic preconditioning during myocardial ischemia-reperfusion. Anesthesiology.

[45] Li S, Xu J, Yao W, Li H, Liu Q, Xiao F, Irwin MG, Xia Z, Ruan W (2015). Sevoflurane pretreatment attenuates TNF-α-induced human endothelial cell dysfunction through activating eNOS/NO pathway. Biochem Biophys Res Commun.

[46] Wang C, Weihrauch D, Schwabe DA, Bienengraeber M, Warltier DC, Kersten JR, Pratt PF, Pagel PS (2006). Extracellular signal-regulated kinases trigger isoflurane preconditioning concomitant with upregulation of hypoxia-inducible factor-1alpha and vascular endothelial growth factor expression in rats. Anesth Analg.

[47] Konia MR, Schaefer S, Liu H (2009). Nuclear factor-[kappa]B inhibition provides additional protection against ischaemia/reperfusion injury in delayed sevoflurane preconditioning. Eur J Anaesthesiol.

[48] Wang C, Xie H, Liu X, Qin Q, Wu X, Liu H, Liu C (2010). Role of nuclear factor-κB in volatile anaesthetic preconditioning with sevoflurane during myocardial ischaemia/reperfusion. Eur J Anaesthesiol.

[49] Tambuyzer BR, Ponsaerts P, Nouwen EJ (2009). Microglia: gatekeepers of central nervous system immunology. J Leukoc Biol.

[50] Wang S, Wang H, Guo H, Kang L, Gao X, Hu L (2011). Neuroprotection of Scutellarin is mediated by inhibition of microglial inflammatory activation. Neuroscience.

[51] Hyakkoku K, Hamanaka J, Tsuruma K, Shimazawa M, Tanaka H, Uematsu S, Akira S, Inagaki N, Nagai H, Hara H (2010). Toll-like receptor 4 (TLR4), but not TLR3 or TLR9, knock-out mice have neuroprotective effects against focal cerebral ischemia. Neuroscience.

[52] Yao L, Kan EM, Lu J, Hao A, Dheen ST, Kaur C, Ling EA (2013). Toll-like receptor 4 mediates microglial activation and production of inflammatory mediators in neonatal rat brain following hypoxia: role of TLR4 in hypoxic microglia. J Neuroinflammation.

[53] Facci L, Barbierato M, Marinelli C, Argentini C, Skaper SD, Giusti P (2014). Toll-like receptors 2, -3 and -4 prime microglia but not astrocytes across central nervous system regions for ATP-dependent interleukin-1β release. Sci Rep.

[54] Xiao Z, Ren P, Chao Y, Wang Q, Kuai J, Lv M, Chen L, Gao C, Sun X (2015). Protective role of isoflurane pretreatment in rats with focal cerebral ischemia and the underlying molecular mechanism. Mol Med Rep.

[55] Marsh BJ, Williams-Karnesky RL, Stenzel-Poore MP (2009). Toll-like receptor signaling in endogenous neuroprotection and stroke. Neuroscience.

[56] Sun M, Deng B, Zhao X, Gao C, Yang L, Zhao H, Yu D, Zhang F, Xu L, Chen L, Sun X (2015). Isoflurane preconditioning provides neuroprotection against stroke by regulating the expression of the TLR4 signalling pathway to alleviate microglial activation. Sci Rep.

[57] Wang H, Lu S, Yu Q, Liang W, Gao H, Li P, Gan Y, Chen J, Gao Y (2011). Sevoflurane preconditioning confers neuroprotection via anti-inflammatory effects. Front Biosci (Elite Ed).

[58] Zhang Y, Tian SY, Li YW, Zhang L, Yu JB, Li J, Chen YY, Wang YX, Liang Y, Zhang XS, Wang WS, Liu HG (2015). Sevoflurane preconditioning improving cerebral focal ischemia-reperfusion damage in a rat model via PI3K/Akt signaling pathway. Gene.

[59] Zhao ZQ, Vinten-Johansen J (2002). Myocardial apoptosis and ischemic preconditioning. Cardiovasc Res.

[60] Broughton BR, Reutens DC, Sobey CG (2009). Apoptotic mechanisms after cerebral ischemia. Stroke.

[61] Deveraux QL, Schendel SL, Reed JC (2001). Antiapoptotic proteins. The bcl-2 and inhibitor of apoptosis protein families. Cardiol Clin.

[62] Zhang T, Saghatelian A (2013). Emerging roles of lipids in BCL-2 family-regulated apoptosis. Biochim Biophys Acta.

[63] Raphael J, Zuo Z, Abedat S, Beeri R, Gozal Y (2008). Isoflurane preconditioning decreases myocardial infarction in rabbits via up-regulation of hypoxia inducible factor 1 that is mediated by mammalian target of rapamycin. Anesthesiology.

[64] Choi H, Kim SH, Chun YS, Cho YS, Park JW, Kim MS (2006). In vivo hyperoxic preconditioning prevents myocardial infarction by expressing bcl-2. Exp Biol Med (Maywood).

[65] Misra A, Haudek SB, Knuefermann P, Vallejo JG, Chen ZJ, Michael LH, Sivasubramanian N, Olson EN, Entman ML, Mann DL (2003). Nuclear factor-kappaB protects the adult cardiac myocyte against ischemia-induced apoptosis in a murine model of acute myocardial infarction. Circulation.

[66] Bedirli N, Bagriacik EU, Emmez H, Yilmaz G, Unal Y, Ozkose Z (2012). Sevoflurane and isoflurane preconditioning provides neuroprotection by inhibition of apoptosis-related mRNA expression in a rat model of focal cerebral ischemia. J Neurosurg Anesthesiol.

[67] Wang H, Shi H, Yu Q, Chen J, Zhang F, Gao Y (2016). Sevoflurane preconditioning confers neuroprotection via anti-apoptosis effects. Acta Neurochir Suppl.

[68] Zhao P, Peng L, Li L, Xu X, Zuo Z (2007). Isoflurane preconditioning improves long-term neurologic outcome after hypoxic-ischemic brain injury in neonatal rats. Anesthesiology.

[69] Codaccioni JL, Velly LJ, Moubarik C, Bruder NJ, Pisano PS, Guillet BA (2009). Sevoflurane preconditioning against focal cerebral ischemia: inhibition of apoptosis in the face of transient improvement of neurological outcome. Anesthesiology.

[70] Bhuiyan MI, Jung SY, Kim HJ, Lee YS, Jin C (2011). Major role of the PI3 K/Akt pathway in ischemic tolerance induced by sublethal oxygen-glucose deprivation in cortical neurons in vitro. Arch Pharm Res.

[71] Chen Y, Nie H, Tian L, Tong L, Deng J, Zhang Y, Dong H, Xiong L (2015). Sevoflurane preconditioning-induced neuroprotection is associated with Akt activation via carboxy-terminal modulator protein inhibition. Br J Anaesth.

[72] Dreier JP (2011). The role of spreading depression, spreading depolarization and spreading ischemia in neurological disease. Nat Med.

[73] Kudo C, Nozari A, Moskowitz MA, Ayata C (2008). The impact of anesthetics and hyperoxia on cortical spreading depression. Exp Neurol.

[74] Bosche B, Graf R, Ernestus RI, Dohmen C, Reithmeier T, Brinker G, Strong AJ, Dreier JP, Woitzik J, Members of the Cooperative Study of Brain Injury Depolarizations (COSBID) (2010). Recurrent spreading depolarizations after subarachnoid hemorrhage decreases oxygen availability in human cerebral cortex. Ann Neurol.

[75] Hartings JA, Bullock MR, Okonkwo DO, Murray LS, Murray GD, Fabricius M, Maas AI, Woitzik J, Sakowitz O, Mathern B, Roozenbeek B, Lingsma H, Dreier JP, Puccio AM, Shutter LA, Pahl C, Strong AJ, Co-Operative Study on Brain Injury Depolarisations (2011). Spreading depolarisations and outcome after traumatic brain injury: a prospective observational study. Lancet Neurol.

[76] Lauritzen M, Dreier JP, Fabricius M, Hartings JA, Graf R, Strong AJ (2011). Clinical relevance of cortical spreading depression in neurological disorders: migraine, malignant stroke, subarachnoid and intracranial hemorrhage, and traumatic brain injury. J Cereb Blood Flow Metab.

[77] Takagaki M, Feuerstein D, Kumagai T, Gramer M, Yoshimine T, Graf R (2014). Isoflurane suppresses cortical spreading depolarizations compared to propofol—implications for sedation of neurocritical care patients. Exp Neurol.

[78] Kudo C, Toyama M, Boku A, Hanamoto H, Morimoto Y, Sugimura M, Niwa H (2013). Anesthetic effects on susceptibility to cortical spreading depression. Neuropharmacology.

[79] De Hert SG, Van der Linden PJ, Cromheecke S, Meeus R, Nelis A, Van Reeth V, ten Broecke PW, De Blier IG, Stockman BA, Rodrigus IE (2004). Cardioprotective properties of sevoflurane in patients undergoing coronary surgery with cardiopulmonary bypass are related to the modalities of its administration. Anesthesiology.

[80] De Hert SG, Cromheecke S, ten Broecke PW, Mertens E, De Blier IG, Stockman BA, Rodrigus IE, Van der Linden PJ (2003). Effects of propofol, desflurane, and sevoflurane on recovery of myocardial function after coronary surgery in elderly high-risk patients. Anesthesiology.

[81] Wang J, Zheng H, Chen CL, Lu W, Zhang YQ (2013). Sevoflurane at 1 MAC provides optimal myocardial protection during off-pump CABG. Scand Cardiovasc J.

[82] Landoni G, Biondi-Zoccai GG, Zangrillo A, Bignami E, D’Avolio S, Marchetti C, Calabrò MG, Fochi O, Guarracino F, Tritapepe L, De Hert S, Torri G (2007). Desflurane and sevoflurane in cardiac surgery: a meta-analysis of randomized clinical trials. J Cardiothorac Vasc Anesth.

[83] Tempe DK, Dutta D, Garg M, Minhas H, Tomar A, Virmani S (2011). Myocardial protection with isoflurane during off-pump coronary artery bypass grafting: a randomized trial. J Cardiothorac Vasc Anesth.

[84] Guerrero Orriach JL, Galán Ortega M, Ramirez Aliaga M, Iglesias P, Rubio Navarro M, Cruz Mañas J (2013). Prolonged sevoflurane administration in the off-pump coronary artery bypass graft surgery: beneficial effects. J Crit Care.

[85] Amr YM, Yassin IM (2010). Cardiac protection during on-pump coronary artery bypass grafting: ischemic versus isoflurane preconditioning. Semin Cardiothorac Vasc Anesth.

[86] Jovic M, Unic-Stojanovic D, Isenovic E, Manfredi R, Cekic O, Ilijevski N, Babic S, Radak D (2015). Anesthetics and cerebral protection in patients undergoing carotid endarterectomy. J Cardiothorac Vasc Anesth.

[87] Zwerus R, Absalom A (2015). Update on anesthetic neuroprotection. Curr Opin Anaesthesiol.

[88] Engelhard K, Werner C (2006). Inhalational or intravenous anesthetics for craniotomies? Pro inhalational. Curr Opin Anaesthesiol.

[89] Holmström A, Akeson J (2005). Sevoflurane induces less cerebral vasodilation than isoflurane at the same A-line autoregressive index level. Acta Anaesthesiol Scand.

[90] Kaisti KK, Långsjö JW, Aalto S, Oikonen V, Sipilä H, Teräs M, Hinkka S, Metsähonkala L, Scheinin H (2003). Effects of sevoflurane, propofol, and adjunct nitrous oxide on regional cerebral blood flow, oxygen consumption, and blood volume in humans. Anesthesiology.

[91] Schoen J, Husemann L, Tiemeyer C, Lueloh A, Sedemund-Adib B, Berger KU, Hueppe M, Heringlake M (2011). Cognitive function after sevoflurane- vs propofol-based anaesthesia for on-pump cardiac surgery: a randomized controlled trial. Br J Anaesth.

[92] Dabrowski W, Rzecki Z, Czajkowski M, Pilat J, Wacinski P, Kotlinska E, Sztanke M, Sztanke K, Stazka K, Pasternak K (2012). Volatile anesthetics reduce biochemical markers of brain injury and brain magnesium disorders in patients undergoing coronary artery bypass graft surgery. J Cardiothorac Vasc Anesth.

[93] Vos PE, Lamers KJ, Hendriks JC, van Haaren M, Beems T, Zimmerman C, van Geel W, de Reus H, Biert J, Verbeek MM (2004). Glial and neuronal proteins in serum predict outcome after severe traumatic brain injury. Neurology.

[94] Ishigaki D, Ogasawara K, Suga Y, Saito H, Chida K, Kobayashi M, Yoshida K, Otawara Y, Ogawa A (2008). Concentration of matrix metalloproteinase-9 in the jugular bulb during carotid endarterectomy correlates with severity of intraoperative cerebral ischemia. Cerebrovasc Dis.

[95] Villa F, Iacca C, Molinari AF, Giussani C, Aletti G, Pesenti A, Citerio G (2012). Inhalation versus endovenous sedation in subarachnoid hemorrhage patients: effects on regional cerebral blood flow. Crit Care Med.

[96] Bösel J, Purrucker JC, Nowak F, Renzland J, Schiller P, Pérez EB, Poli S, Brunn B, Hacke W, Steiner T (2012). Volatile isoflurane sedation in cerebrovascular intensive care patients using AnaConDa(^®^): effects on cerebral oxygenation, circulation, and pressure. Intensive Care Med.

[97] Chui J, Mariappan R, Mehta J, Manninen P, Venkatraghavan L (2014). Comparison of propofol and volatile agents for maintenance of anesthesia during elective craniotomy procedures: systematic review and meta-analysis. Can J Anaesth.

[98] Ishida K, Berger M, Nadler J, Warner DS (2014). Anesthetic neuroprotection: antecedents and an appraisal of preclinical and clinical data quality. Curr Pharm Des.

[99] Xu Z, Yu J, Wu J, Qi F, Wang H, Wang Z, Wang Z (2016). The effects of two anesthetics propofol and sevoflurane, on liver ischemia/reperfusion injury. Cell Physiol Biochem.

[100] Wu Y, Gu C, Huang X (2016). Sevoflurane protects against hepatic ischemia/reperfusion injury by modulating microRNA-200c regulation in mice. Biomed Pharmacother.

[101] Ohsumi A, Marseu K, Slinger P, McRae K, Kim H, Guan Z, Hwang DM, Liu M, Keshavjee S, Cypel M (2017). Sevoflurane attenuates ischemia-reperfusion injury in a rat lung transplantation model. Ann Thorac Surg.

[102] Guye ML, Mc Gregor B, Weil G, Arnal F, Piriou V (2010). Ischaemic and pharmacologic preconditioning: desflurane reduces renal reperfusion injury in rabbits. Ann Fr Anesth Reanim.

[103] Lucchinetti E, Ambrosio S, Aguirre J, Herrmann P, Härter L, Keel M, Meier T, Zaugg M (2007). Sevoflurane inhalation at sedative concentrations provides endothelial protection against ischemia-reperfusion injury in humans. Anesthesiology.

[104] Garcia C, Julier K, Bestmann L, Zollinger A, von Segesser LK, Pasch T, Spahn DR, Zaugg M (2005). Preconditioning with sevoflurane decreases PECAM-1 expression and improves one-year cardiovascular outcome in coronary artery bypass graft surgery. Br J Anaesth.

[105] Kharbanda RK, Peters M, Walton B, Kattenhorn M, Mullen M, Klein N, Vallance P, Deanfield J, MacAllister R (2001). Ischemic preconditioning prevents endothelial injury and systemic neutrophil activation during ischemia-reperfusion in humans in vivo. Circulation.

[106] Jambrik Z, Santarcangelo EL, Rudisch T, Varga A, Forster T, Carli G (2005). Modulation of pain-induced endothelial dysfunction by hypnotisability. Pain.

[107] Sanada H, Higashi Y, Goto C, Chayama K, Yoshizumi M, Sueda T (2005). Vascular function in patients with lower extremity peripheral arterial disease: a comparison of functions in upper and lower extremities. Atherosclerosis.

